# Resveratrol Ameliorates Imiquimod-Induced Psoriasis-Like Skin Inflammation in Mice

**DOI:** 10.1371/journal.pone.0126599

**Published:** 2015-05-12

**Authors:** Thomas Nordstrøm Kjær, Kasper Thorsen, Niels Jessen, Karin Stenderup, Steen Bønløkke Pedersen

**Affiliations:** 1 Department of Endocrinology and Internal medicine, Aarhus University Hospital, Aarhus, Denmark; 2 Department of Clinical Medicine, Aarhus University, Aarhus, Denmark; 3 Department of Molecular Medicine, Aarhus University Hospital, Aarhus, Denmark; 4 Department of Dermatology, Aarhus University Hospital, Aarhus, Denmark; INSERM-Université Paris-Sud, FRANCE

## Abstract

**Background:**

The polyphenol resveratrol has anti-inflammatory effects in various cells, tissues, animals and human settings of low-grade inflammation. Psoriasis is a disease of both localized and systemic low-grade inflammation. The Sirtuin1 enzyme thought to mediate the effects of resveratrol is present in skin and resveratrol is known to down regulate NF-κB; an important contributor in the development of psoriasis. Consequently we investigated whether resveratrol has an effect on an Imiquimod induced psoriasis-like skin inflammation in mice and sought to identify candidate genes, pathways and interleukins mediating the effects.

**Methods:**

The study consisted of three treatment groups: A control group, an Imiquimod group and an Imiquimod+resveratrol group. Psoriasis severity was assessed using elements of the Psoriasis Area Severity Index, skin thickness measurements, and histological examination. We performed an RNA microarray from lesional skin and afterwards Ingenuity pathway analysis to identify affected signalling pathways. Our microarray was compared to a previously deposited microarray to determine if gene changes were psoriasis-like, and to a human microarray to determine if findings could be relevant in a human setting.

**Results:**

Imiquimod treatment induced a psoriasis-like skin inflammation. Resveratrol significantly diminished the severity of the psoriasis-like skin inflammation. The RNA microarray revealed a psoriasis-like gene expression-profile in the Imiquimod treated group, and highlighted several resveratrol dependent changes in relevant genes, such as increased expression of genes associated with retinoic acid stimulation and reduced expression of genes involved in IL-17 dependent pathways. Quantitative PCR confirmed a resveratrol dependent decrease in mRNA levels of IL-17A and IL-19; both central in developing psoriasis.

**Conclusions:**

Resveratrol ameliorates psoriasis, and changes expression of retinoic acid stimulated genes, IL-17 signalling pathways, IL-17A and IL-19 mRNA levels in a beneficial manner, which suggests resveratrol, might have a role in the treatment of psoriasis and should be explored further in a human setting.

## Introduction

Psoriasis is a common chronic skin disease with a prevalence of 0.6–4.8%[1]. An exact cause of psoriasis has not yet been established, but genetic predisposition and external stimuli e.g. stress, infection, trauma, and drugs are thought to be the culprits.

Psoriasis is an inflammatory disease with increased expression of pro-inflammatory cytokines and chemokines attracting immune cells to the psoriatic skin area, where a proliferation of local and invading cells takes place [2]. However, psoriasis is also a disease of systemic low-grade inflammation, which might be the link to co-morbidities like cardiovascular disease and the metabolic syndrome [3–5]. In short, a current disease model is as follows: Keratinocytes release pro-inflammatory cytokines such as interleukin (IL)-6, IL-1β and tumour necrosis factor alpha (TNFα) when under stress. This triggers plasmacytoid dendritic cells into producing and secreting interferon(IFN)-α. IFN-α activates dermal myeloid dendritic cells which migrate to local lymph nodes and secrete IL-12 and IL-23. IL-12 and IL-23 in turn activate circulating naive T-helper lymphocytes (type 1, 17 and 22). These lymphocytes migrate to the skin and secrete IFN-γ, IL-17A, IL-17F, IL-22 and thus partake in an intricate immunological crosstalk between the local and invading cells, driving an uncontrolled inflammation and stimulus for keratinocyte hyperproliferation [2, 6].

Interestingly several of these pro-inflammatory cytokines e.g. TNFα, IL-12 and IL-23 rely on nuclear factor kappa B (NF-κB) as a downstream mediator of their effects on a transcriptional level. Accordingly, increased levels of activated NF-κB are found in psoriasis skin compared with healthy skin [2, 7].

Resveratrol (RSV) is a compound found in grapes, nuts and berries and it possesses anti-inflammatory effects in macrophage cell lines, adipocyte cell lines, cultured adipocytes and adipose tissue. We have found that human adipocytes and stromal-vascular cells from adipose tissue express Sirtuin 1 enzyme (SIRT1), which is thought to be an essential component in the RSV pathway[8]. SIRT1 is present in skin and is thought to inhibit proliferation and promote differentiation [9]. Furthermore, we have found that RSV has strong anti-inflammatory effects in human adipose tissue explants [10, 11] and in part this might be a result of down regulation of NF-κB [12]. Despite these profound effects of RSV on cellular metabolism RSV is very well tolerated in cultured primary cells, cell lines and RSV has been used in a human clinical trial with few and only mild side effects[13].

Treatment of psoriasis comprises a wide range of options from topical treatments (e.g. local steroid and vitamin D analogues), to heliotherapy (e.g. UVB and PUVA), systemic treatment (e.g. methotrexate, acitretin and cyclosporine), and biological treatment (e.g. anti-TNFα and anti-IL-12/23 antibodies) [14]. Some of these treatments can cause severe side effects, and therefore safer treatment modalities would be valuable in the management of psoriasis. Owing to the fact that RSV possesses strong anti-inflammatory effects in various cells and tissues, the SIRT1 enzyme is present in skin and NF-κB is important in the development of psoriasis, we decided to study whether RSV might possess positive effects on Imiquimod (IMQ)-induced psoriasis-like skin inflammation in the mouse model described by van der Fits *et al*. [15] and furthermore, identify potential candidate genes, pathways and interleukins responsible for the observed effect.

## Materials and Methods

### Animals and Ethical Statement

Twenty-eight male BALBc/AnNTac mice 6–7 weeks of age (Taconic, Ry, Denmark) were kept in cages at constant levels of temperature and humidity on 12-hour light/dark cycles. The animals had their backs shaved and were allowed 4 days of acclimatization before any experimental procedures. The animals had access to unlimited amounts of water and feed during the entire trial.

The Animal Experiments Inspectorate under the authority of the Danish Ministry of Food, Agriculture and Fisheries, approved all experimental procedures and ethical aspects of this study (Application no. 2012-DY-2934-00019) and all experimental procedures were conducted in accordance with the guidelines of the Animal Experiments Inspectorate.

### Study design and treatment

The mice were distributed into 3 groups; a control group, an IMQ group and an IMQ-RSV (8, 10 and 10 mice per group). The mice in groups IMQ and IMQ-RSV received a daily dose of 62.5 mg of 5% IMQ cream (Aldara; MEDA AS) applied on their backs and right ear folds. The mice in the control group received a similar daily dose of vehicle cream (Vaseline Lanette cream; Fagron). The feed was pulverized (standard chow with protein, carbohydrate and fat accounting for 20%/70%/10% of caloric intake, respectively). Trans-RSV was added to the pulverized feed given to the IMQ-RSV group in an amount of 400 mg/kg animal/day based on average food intake [16]. All animals were assessed for the severity of the psoriasis-like skin condition on days 0, 2, 4 and 7, using 2 elements of the Psoriasis Area Severity Index (PASI), to assign a score of 0–4 (0, none; 1, mild; 2, moderate; 3, severe; 4, very severe) for each of the parameters erythema and scaling. Omitting evaluation of induration by PASI, we used thickness of a skinfold on the back (day 7) and thickness of the right ear fold (day 0 and 7) measured by using a calliper (accuracy: ± 0,02mm, Mitsutomo, Japan).

On day 7, the animals were euthanized and the shaved area of skin on their backs was immediately excised. A 4 mm punch biopsy of lesional skin was fixed in formalin and paraffin embedded for histological analysis. The remaining lesional skin was snap frozen in liquid nitrogen and stored at -80 C°.

### Histological analyses

The paraffin embedded punch biopsies were sectioned and haematoxylin and eosin (HE) stained for histological evaluation. Blinded to treatment group, epidermal thickness, an accepted end-point for measuring psoriasis severity, was measured as an average of 15–20 random measurements of the distance from the stratum corneum to the deepest part of the epidermis, employing LEICA IM50 software, version 4.0.

### RNA isolation and qPCR analysis

20 mg of skin was used for RNA extraction using TRIzol (Gibco BRL, Life Technologies, Roskilde, Denmark) and homogenized with one tungsten bead (Qiagen Nordic, Copenhagen, Denmark) using a Mixer Mill 300 (Retsch, Haan, Germany). RNA was quantified by measuring absorbance at 260 and 280 nm with a ratio between RNA and protein ≥1.9 using a NanoDrop 8000 Spectrophotometer (Thermo Scientific Pierce, Waltham, Maine, USA). Integrity of the RNA was checked by visual inspection of 18S and 28S ribosomal RNAs on an agarose gel, and a subset of RNA samples were used for RNA microarray analysis (three controls, two IMQs and two IMQ-RSVs). Prior to the analyses, the quality of RNA from all animals was assessed by determining the RNA Quality indicator (RQI number) (all samples had an RQI number> 8) using a Bio-Rad Experion Automated Electrophoresis Station (Bio-Rad Laboratories, Hercules, CA 94547, USA).

Complementary DNA (cDNA) was synthesized using random hexamer primers using the Verso cDNA kit (Applied Biosystems). Quantitative PCR (qPCR) for target genes were performed using MYO18B as a reference gene, which was selected based on RNA microarray analysis as a constant gene in the groups. The stable expression pattern was verified by qPCR and it was similarly expressed in the three groups (ct value in Control group = 28.6 ± 1.8 in IMQ group = 29.0 ± 1.6 and IMQ+RSV group = 29.1 ± 1.6). Sequences of the used primers are shown in S1 Table.

The PCR reactions were performed in duplicates using the KAPA SYBR FAST qPCR kit (Kapa Biosystems, Inc., Woburn, MA) in a LightCycler 480 (Roche Applied Science) using the following protocol: One step at 95°C for 3 min, then 95°C for 10 s, 60°C for 20 s, and 72°C for 10 s. The increase in fluorescence was measured in real time during the extension step and a final melt curve analysis was performed to verify the specificity of the amplification. The relative gene expression was estimated using the default “Advanced Relative Quantification” mode of the software version LCS 480 1.5.0.39 (Roche Applied Science). The specificity of the primers was tested and all had an amplification efficiency of 1.9 to 2.1.

### RNA microarray and pathway analysis

The labelling of 100 ng total RNA was performed using the Ambion WT Expression Kit (Ambion) followed by hybridization to the GeneChip Mouse Gene 1.0 ST Arrays (Affymetrix) according to the manufacturer’s instructions. Scanning was performed in an Affymetrix GCS 3000 7G scanner. RMA16 Quantile normalization, hierarchical clustering, and fold change calculations were performed using the GeneSpring 12.6 software package (Agilent). Transcript IDs, predicted to hybridize to multiple targets, were omitted leaving 25679 transcript IDs for further analysis. Hierarchical clustering was done with Pearson centered distance and average linkage. Unpaired t-test was used for calculating p-values. Microarray data were deposited in the GEO archive under accession number GSE63684.

Gene expression data from an IMQ induced psoriasis mouse model (GSE27628) and human psoriatic samples (GSE13355) were downloaded from GEO. Both datasets were RMA normalized using GeneSpring and MAS5 data analysis was used to filter out non-expressed probesets (present call in no samples).

The GSE27628 dataset contained triplicate samples from lesional skin from the imiquimod-treated mice and the control mice; however, sample GSM684684 was an outlier and removed from the analysis.

The GSE13355 dataset contained biopsies from 58 psoriatic patients (both from involved and uninvolved skin) and 64 normal healthy controls. For the cluster analyses only normal healthy controls and psoriatic involved skin was used. Data comparison across platforms and species was performed in GeneSpring using the translation function that uses the HomoloGene system to find homologs among eukaryotic gene sets.

Ingenuity Pathway Analysis software (Ingenuity Systems) was used for pathway analysis performed with following settings: Species = Mouse AND (confidence = Experimentally Observed OR High (predicted)) AND (tissues = Epidermis OR Dermis OR Skin) AND(data sources = An Open Access Database of Genome-wide Association Results OR BIND OR BIOGRID OR Catalogue Of Somatic Mutations In Cancer (COSMIC) OR Chemical Carcinogenesis Research Information System (CCRIS) OR ClinicalTrials.gov OR ClinVar OR Cognia OR DIP OR DrugBank OR Gene Ontology (GO) OR GVK Biosciences OR Hazardous Substances Data Bank (HSDB) OR HumanCyc OR Ingenuity Expert Findings OR Ingenuity ExpertAssist Findings OR INTACT OR Interactome studies OR MINT OR MIPS OR miRBase OR miRecords OR Mouse Genome Database (MGD) OR Obesity Gene Map Database OR Online Mendelian Inheritance in Man (OMIM) OR TarBase OR TargetScan Human)

### Statistical analysis

Normality was checked by Shapiro-Wilk test and equal variance was by tested by variance ratio test. If appropriate data was log 10 transformed before statistical procedures. Differences between groups were analysed by using one way ANOVA or ANOVA on Ranks. If appropriate after one way ANOVA analysis, post hoc multiple pairwise comparisons were performed using either the Holm-Sidak test or Dunn’s method. If not otherwise stated, results are presented as group means ± standard error of the mean (SEM) and unadjusted P values. Messenger RNA expression is presented as a P-value only.

P values < 0.05 were considered statistically significant in the ANOVA and the overall significance level for post hoc testing = 0.05. All analyses were performed using Sigmaplot 11 (Systator software, Inc., Richmond, CA, USA).

## Results

### Erythema and scaling

During the 7-day trial, the severity of the psoriasis-like skin condition was scored on days 0, 2, 4 and 7 using the erythema and scaling parts of the Psoriasis Area Severity Index (PASI). The psoriasis-like skin conditions became apparent on day 2 and onwards. At no point in time was a psoriasis-like skin condition seen on the backs of the mice treated with vehicle cream.

**Fig 1:**
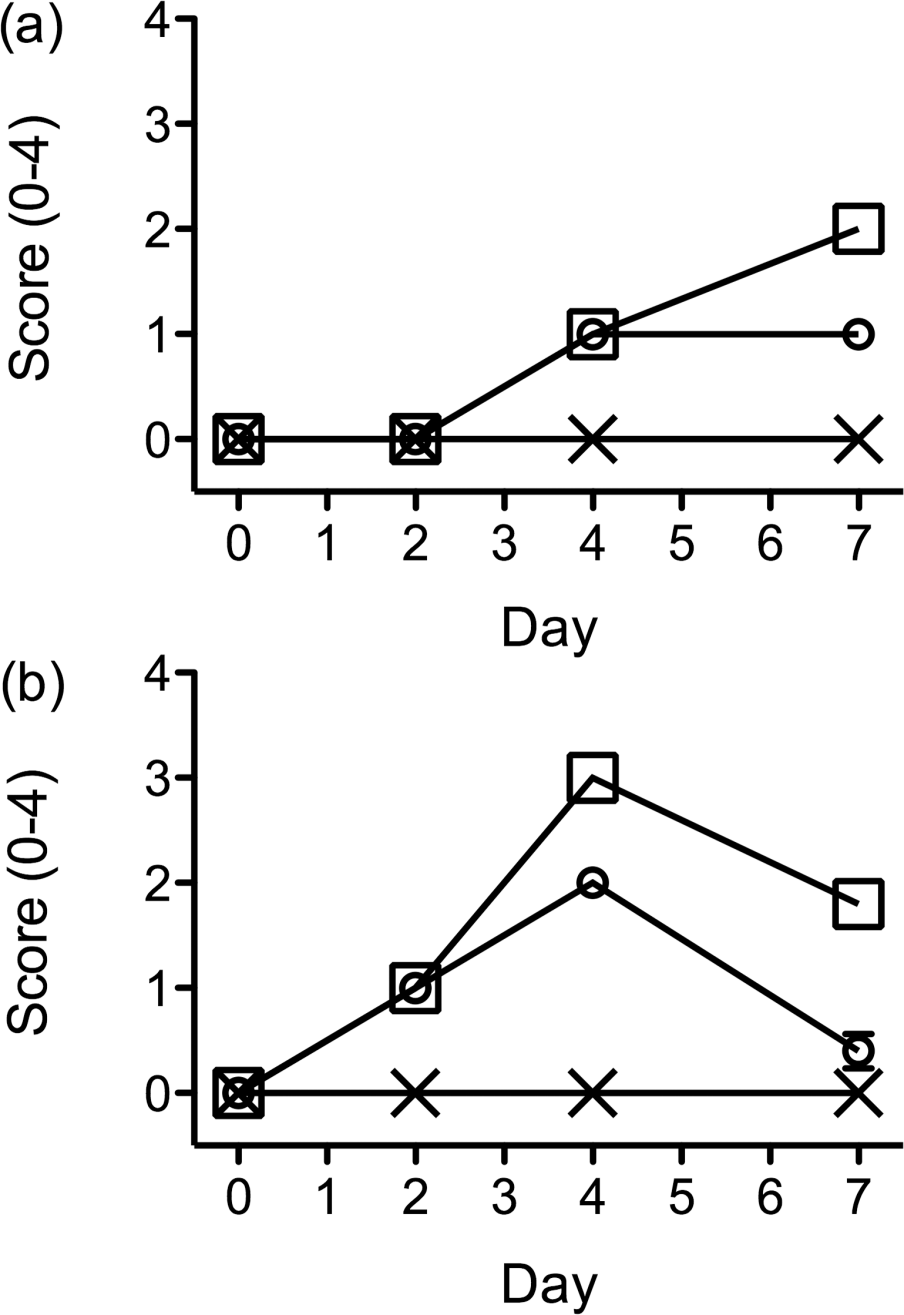
Erythema and scales score of the skin on the backs BALB/c mice. Scoring was performed on days 0, 2, 4 and 7 using the erythema and scales elements of the Psoriasis Area Severity Index (PASI) to assign a score of 0–4 to each animal and thereby assess the effects of daily treatment with Imiquimod cream and vehicle cream. (a) Erythema score: Data points are presented as group means ±SEM (n = 8, n = 10, n = 10 for controls, IMQ and IMQ-RSV respectively) (X = control group, O = IMQ-RSV group, □ = IMQ group). (b) Scales score: Data points are presented as group means ±SEM (n = 8, n = 10, n = 10 for controls, IMQ, IMQ-RSV respectively) (X = control group, O = IMQ-RSV group, □ = IMQ group).

The means of the scores with SEM for erythema and scales is shown for each of the groups on days 0, 2, 4 and 7 (Fig 1).

### Skinfold and ear thickness

**Fig 2:**
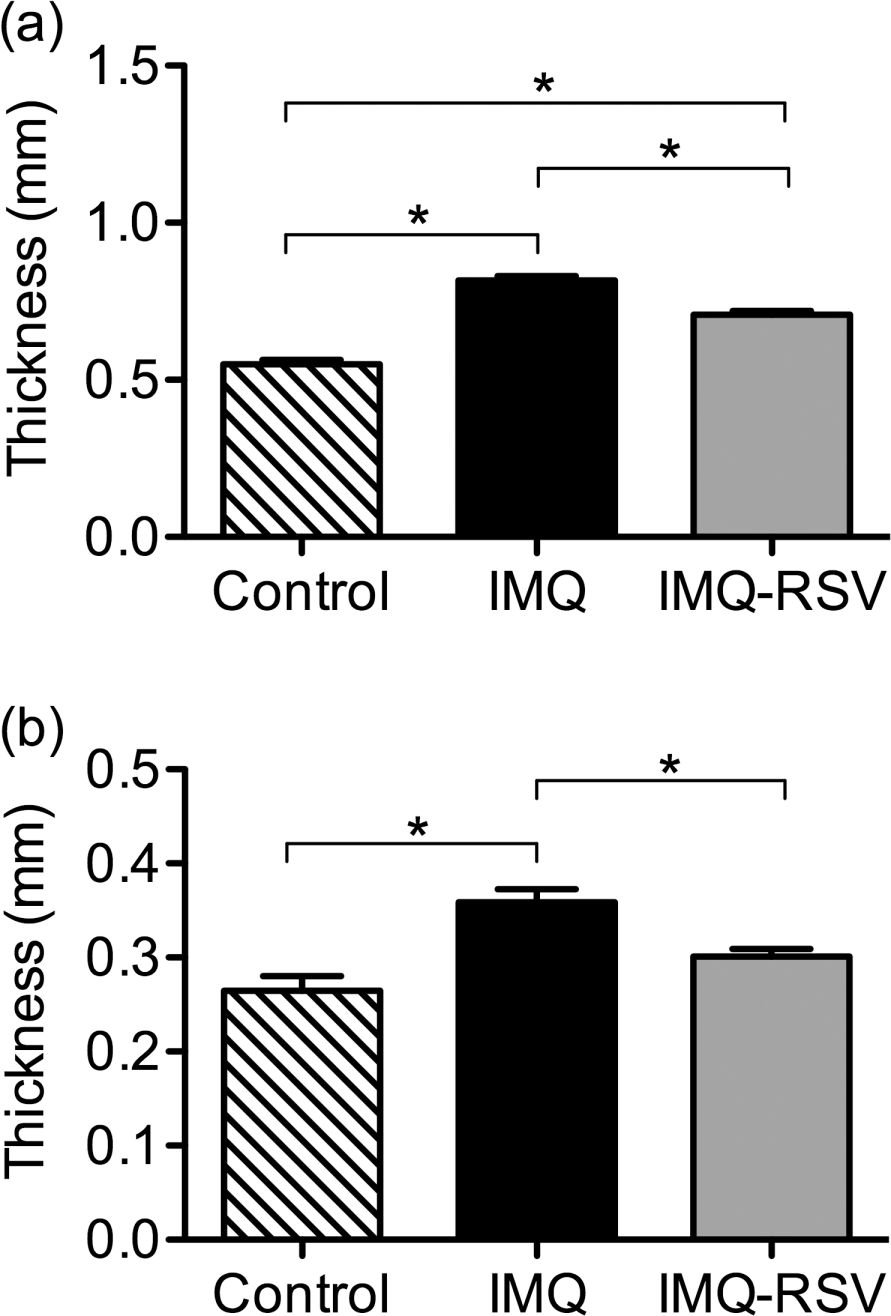
Calliper measurement of skin thickness. The right ear fold and the skinfold on the backs of the mice were measured to quantify the thickening of the skin caused by Imiquimod treatment. (a) Skinfold thickness on the backs of the mice. (b) Right ear fold thickness. Columns represent group means ±SEM of skin/ear fold measurements day 7 ((n = 8, n = 10, n = 10 for controls, IMQ, IMQ-RSV respectively). Clamped bar with * above indicates the pair of column means are significantly different (p<0.05).

Skinfold thickness on the backs of the mice in the IMQ group was significantly increased by IMQ treatment compared with subjects from the control group (0.816mm ±0.0142 vs. 0.550mm ±0.0136; p<0.001). The skinfold thickness in the IMQ-RSV group was significantly reduced compared to the IMQ group (0.816mm ±0.0142 vs. 0.707mm ±0.0121; p<0.001). However, skinfold thickness in the IMQ-RSV group was not completely normalized when compared to controls (0.707mm ±0.0121 vs. 0.550mm ±0.0136; p<0.001) (Fig 2A). Additionally, we measured the thickness of the right ears of the mice in the IMQ group, which were significantly increased compared to those in the control group (0.359mm±0.0135 vs. 0.265mm±0.0151; p<0.001). In the IMQ-RSV group, thickening of the ear was significantly reduced compared to the IMQ group (0.301mm ±0.00795 vs. 0.359mm ±0.0135; p = 0.002). In fact, ear thickness in the IMQ-RSV group was reduced to the point of no ascertainable difference in ear thickness when compared to the control group (0.301 ±0.00795 vs. 0.265mm ±0.0151; p<0.053) (Fig 2B).

### Histological results

**Fig 3:**
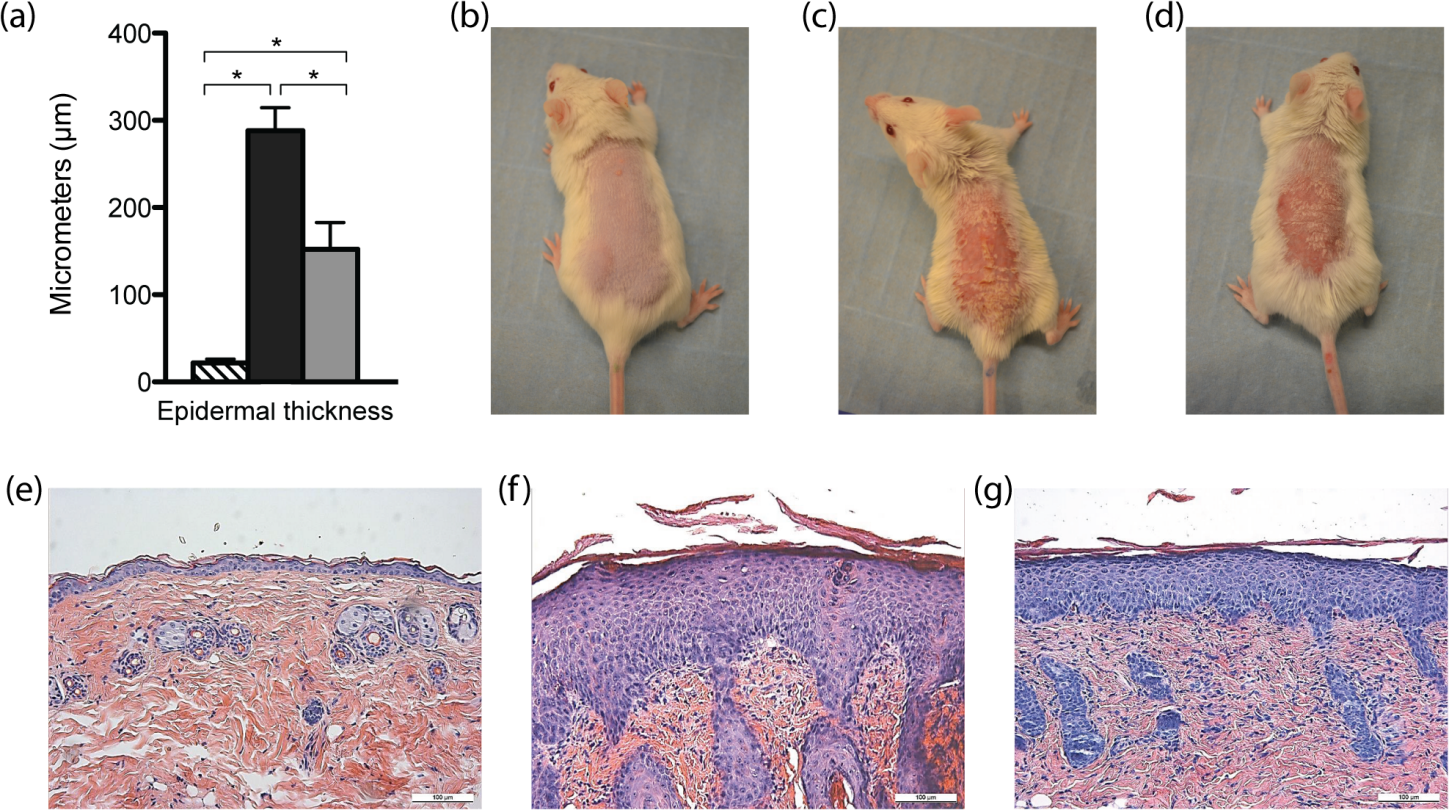
Epidermal thickness measured in skin sections, presentation of the mouse phenotype and HE sectioned skin. (a) Epidermal thickness; Means of epidermal thickness was calculated based on 15–20 random site measurements. (b-d) Presentation of phenotype of mice from control, IMQ and IMQ-RSV groups, respectively. Photograph is taken after 5 days of treatment. (e-g) HE-stained skin sections from the backs of the mice. Sections were used for evaluation of epidermal thickness. In the lower right corner of photos the white box = 100μm. Columns in a) are group means ±SEM (n = 7, n = 5, n = 5 for controls, IMQ, IMQ-RSV respectively). Clamped bar with * above indicates the pair of column means are significantly different (p<0.05). (Symbols: Striped fill = control, black fill = IMQ, grey fill = IMQ-RSV).

Both the IMQ group (n = 5) and the IMQ-RSV group (n = 5) had a significantly thickened epidermis compared with the control group (n = 7) (288μm ±26.5 and 152μm ±30.8, respectively vs. 21.9μm ±4.29; both P<0.001). However, epidermal thickness was significantly reduced in the IMQ-RSV group when compared with the IMQ group (288μm ±26.5 vs. 152μm ±30.8; p<0.001) (Fig 3).

### Gene Expression analysis

The RNA microarray analysis revealed that IMQ treatment induced a profound change in gene expression compared with the control group with 1923 probe sets having a p-value <0.05 and fold change >1.5 (844 up-regulated and 1079 down-regulated). When comparing our gene expression data with the data from another IMQ induced psoriasis mouse model described by Swindell et *al*.[17], we found a good correlation between the two sets of data. Of the 844 up-regulated probe sets, 87% of the expressed probe sets were found to be up-regulated in the Swindell dataset. Of the 1079 down-regulated probe sets, 75% of the expressed probe sets were found to be down-regulated in the Swindell dataset.

To determine the effect of RSV treatment, we chose 244 probe sets with a fold change>1.5 between the IMQ and IMQ-RSV groups. To investigate the clinical relevance of the changes induced in the RSV mice we looked at previously published human psoriatic samples [18]. The 244 probe sets that differ between IMQ and IMQ-RSV in our study correspond to 381 probe sets on the human U133 v2 array of which 330 probe sets were expressed in at least one sample. We used the 330 corresponding expressed human probe sets to cluster 122 patient samples from the Gudjonsson data set [18] and found a perfect separation of these samples into 58 psoriatic and 64 normal patient samples, indicating that these genes may play a role in the human phenotype as well (S1 Fig).

Several genes were chosen for confirmation using qPCR, and there was a good match between changes detected by RNA microarray and qPCR (Pearson correlation = 0.7, p<0.001) (S2 Table).

**Fig 4:**
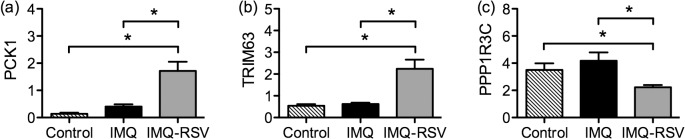
Quantitative PCR of microarray genes. Selected qPCR of genes that were 1.5 fold or more changed by RSV treatment in the microarray (RSV treated compared with the IMQ group). The mRNA levels were quantified using MYO18B as reference gene. (a) Phosphoenolpyruvate Carboxykinase 1 (PCK1). (b) Tripartite Motif Containing 63, E3 Ubiquitin Protein Ligase (TRIM63). (c) Protein Phosphatase 1, Regulatory (inhibitor) Subunit 3C (PPP1R3C). Columns in (a-c) are group means ±SEM (n = 8, n = 10, n = 10 for controls, IMQ, IMQ-RSV respectively). Clamped bar with * above indicates the pair of column means are significantly different (p<0.05).(Symbols: Striped fill = control, black fill = IMQ, grey fill = IMQ-RSV).

As shown in Fig 4 RSV induced a significant increase in Phosphoenolpyruvate Carboxykinase 1 (PCK1), and Tripartite Motif Containing 63, E3 Ubiquitin Protein Ligase (TRIM63), whereas Protein Phosphatase 1, Regulatory (inhibitor) Subunit 3C (PPP1R3C) was significantly decreased.

**Fig 5:**
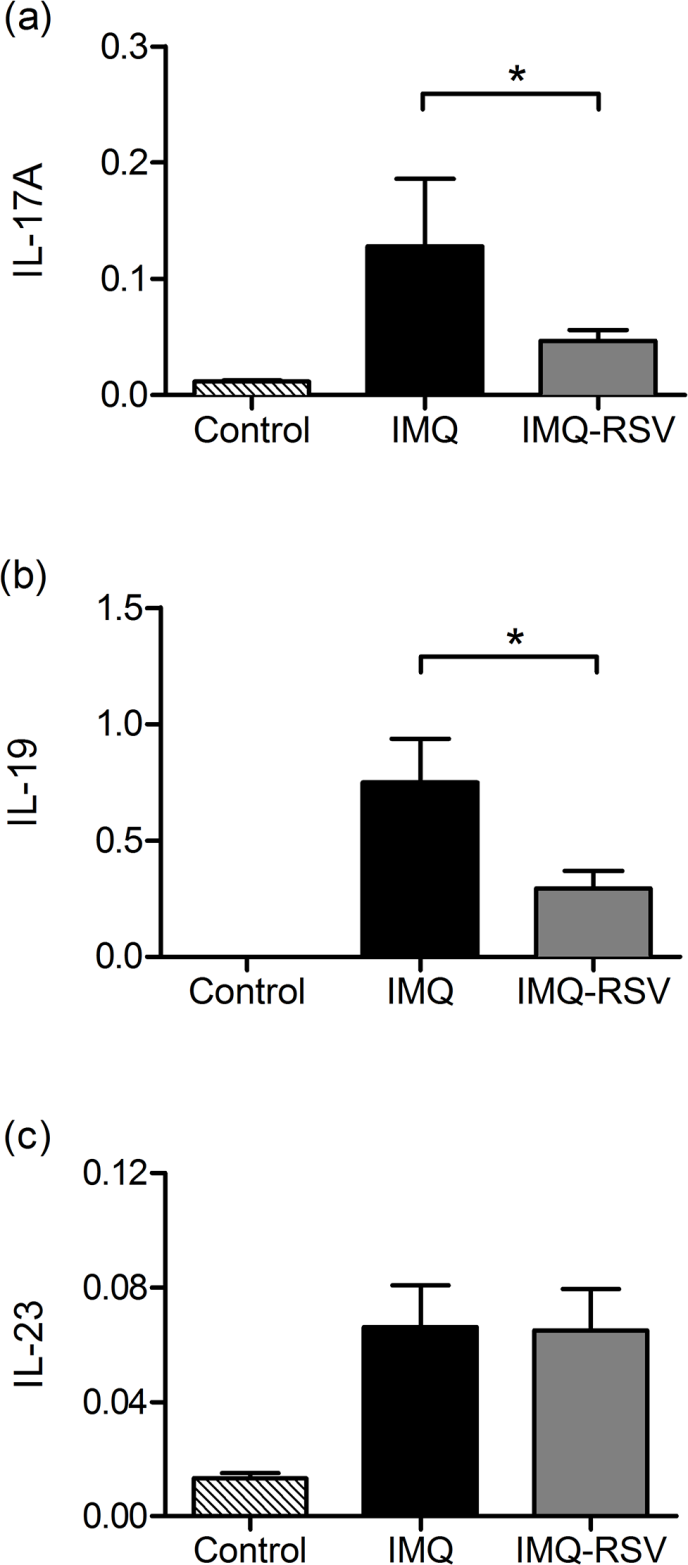
RSV effects on IL-17A, IL-19 and IL-23p19 gene expression. Quantitative PCR of IL-17A, IL-19 and IL-23p19 gene expression was determined to quantify effects of RSV on IL-17A, IL-19 and IL-23p19 gene expression. The mRNA levels of IL-17A, IL-19 and IL-23p19 were quantified using MYO18B as reference gene. Clamped bar with * above indicates the pair of column means are significantly different (p<0.05). Striped fill = control, black fill = IMQ, grey fill = IMQ-RSV.

The RNA microarray analysis indicated an increased gene expression of IL-19, IL-17A, and IL-23p19 in the IMQ treated skin compared with control samples, and RSV treatment resulted in a lower gene expression of IL-19, IL-17A and IL-23p19 compared to IMQ treated skin. Subsequent qPCR validation of IL-17A, IL-19 and IL-23p19 gene expression confirmed that IMQ significantly increased mRNA expression of IL-17A, IL-19 IL-23p19 in the IMQ group compared to the control group. RSV treatment significantly reduced mRNA expression of IL-17A and IL-19 compared to the IMQ group (p = 0.018 and p = 0.047 respectively) (Fig 5) whereas qPCR could not confirm a reduction in IL-23p19 after RSV treatment (Fig 5). A similar pattern was seen for TNFα, where RSV treatment was unable to reduce the TNFα expression compared with the IMQ treatment group (data not shown).

Using Ingenuity Pathways Analysis software to characterize the significant differences between IMQ and IMQ-RSV groups, we found that RSV influenced the pathways shown in Table 1. Several pathways are of relevance for studying the effects of RSV in psoriasis e.g. Retinoic X Receptor (RXR) and IL-17 dependent pathways.

**Table 1:** Significantly RSV changed pathway.

Ingenuity Canonical Pathways	-log(p-value)	Ratio
Cholecystokinin/Gastrin-mediated Signaling	3,66E00	4,92E-02
Glucocorticoid Receptor Signaling	3,62E00	2,47E-02
PPAR Signaling	3,62E00	4,76E-02
LXR/RXR Activation	3,54E00	4,48E-02
Differential Regulation of Cytokine Production in Intestinal Epithelial Cells by IL-17A and IL-17F	3,35E00	1,25E-01
IL-6 Signaling	3,33E00	3,8E-02
Hepatic Cholestasis	3,17E00	3,37E-02
Granulocyte Adhesion and Diapedesis	3,12E00	3,23E-02
Role of Hypercytokinemia/hyperchemokinemia in the Pathogenesis of Influenza	3,07E00	9,09E-02
Agranulocyte Adhesion and Diapedesis	3,04E00	3,03E-02
Graft-versus-Host Disease Signaling	3E00	8,33E-02
Role of Cytokines in Mediating Communication between Immune Cells	2,96E00	8E-02
Role of IL-17A in Arthritis	2,74E00	6,25E-02
TREM1 Signaling	2,64E00	5,56E-02
Communication between Innate and Adaptive Immune Cells	2,51E00	4,76E-02
IL-10 Signaling	2,41E00	4,26E-02
FXR/RXR Activation	2,4E00	4,17E-02
Altered T Cell and B Cell Signaling in Rheumatoid Arthritis	2,38E00	4,08E-02
Toll-like Receptor Signaling	2,36E00	4E-02
Role of Tissue Factor in Cancer	2,13E00	3,03E-02
Atherosclerosis Signaling	2,08E00	2,86E-02
p38 MAPK Signaling	2,06E00	2,82E-02
Pancreatic Adenocarcinoma Signaling	2,05E00	2,78E-02
Pregnenolone Biosynthesis	2E00	2E-01
Airway Pathology in Chronic Obstructive Pulmonary Disease	1,92E00	1,67E-01
Role of IL-17A in Psoriasis	1,92E00	1,67E-01
Histidine Degradation VI	1,92E00	1,67E-01
Dendritic Cell Maturation	1,86E00	2,2E-02
Systemic Lupus Erythematosus Signaling	1,86E00	2,2E-02
Prostanoid Biosynthesis	1,85E00	1,43E-01
Ubiquinol-10 Biosynthesis (Eukaryotic)	1,8E00	1,25E-01
Acute Phase Response Signaling	1,79E00	2,02E-02
NF-κB Signaling	1,7E00	1,82E-02
Fatty Acid α-oxidation	1,7E00	1E-01
LPS/IL-1 Mediated Inhibition of RXR Function	1,69E00	1,79E-02
IL-8 Signaling	1,62E00	1,65E-02
Differential Regulation of Cytokine Production in Macrophages and T Helper Cells by IL-17A and IL-17F	1,56E00	7,14E-02
Hepatic Fibrosis / Hepatic Stellate Cell Activation	1,54E00	1,49E-02
Role of Osteoblasts, Osteoclasts and Chondrocytes in Rheumatoid Arthritis	1,49E00	1,41E-02
MIF-mediated Glucocorticoid Regulation	1,4E00	5E-02
Docosahexaenoic Acid (DHA) Signaling	1,33E00	4,17E-02
MIF Regulation of Innate Immunity	1,31E00	4E-02
IL-17A Signaling in Fibroblasts	1,31E00	4E-02
Role of IL-17F in Allergic Inflammatory Airway Diseases	1,31E00	4E-02
Role of Macrophages, Fibroblasts and Endothelial Cells in Rheumatoid Arthritis	1,3E00	1,1E-02
Eicosanoid Signaling	1,18E00	2,94E-02
IL-17A Signaling in Airway Cells	1,17E00	2,86E-02
CD40 Signaling	1,14E00	2,7E-02
IL-17 Signaling	1,1E00	2,44E-02
Role of MAPK Signaling in the Pathogenesis of Influenza	1,09E00	2,38E-02
Small Cell Lung Cancer Signaling	1,05E00	2,17E-02
G Beta Gamma Signaling	1,02E00	2E-02
ErbB Signaling	1,02E00	2E-02
Neuregulin Signaling	9,79E-01	1,82E-02
HGF Signaling	9,23E-01	1,59E-02
Type I Diabetes Mellitus Signaling	8,92E-01	1,47E-02
Role of Pattern Recognition Receptors in Recognition of Bacteria and Viruses	8,86E-01	1,45E-02
Corticotropin Releasing Hormone Signaling	8,86E-01	1,45E-02
PI3K/AKT Signaling	8,63E-01	1,37E-02
HMGB1 Signaling	8,36E-01	1,28E-02
Ovarian Cancer Signaling	8,36E-01	1,28E-02
Aryl Hydrocarbon Receptor Signaling	7,88E-01	1,14E-02
Endothelin-1 Signaling	7,88E-01	1,14E-02
PPARα/RXRα Activation	7,37E-01	1E-02
ILK Signaling	7,07E-01	9,26E-03
Xenobiotic Metabolism Signaling	6,38E-01	7,75E-03
Colorectal Cancer Metastasis Signaling	6,1E-01	7,19E-03
Protein Kinase A Signaling	4,83E-01	5,1E-03

List of RSV dependent pathway changes reaching statistical significance. Probe sets with fold change> 1.5 were analysed and a p-value < 0.05 was considered statistically significant in the analysis. The list is ranked by p-value from lowest to highest. Pathway analysis was performed using Ingenuity Pathway Analysis software.

## Discussion

We have previously shown that RSV has anti-inflammatory effects in several cell lines and human tissues [10, 11]. Activation of NF-κB is a key factor in the development of psoriasis [7] and in a previous study, RSV has been shown to inhibit NF-κB [12] and decrease NF-κB activity in cultured keratinocytes exposed to LPS, TNFα and IFN-γ [19].These findings and the fact that SIRT1 one is present in skin led us to test whether or not RSV *in vivo* would have an effect on psoriasis-like skin inflammation in a mouse model.

By using the IMQ model described by van der Fits *et al*.[15], we induced a psoriasis-like skin condition as seen by increased skin thickness measured by the use of a calliper, as well as an increased PASI score for erythema and scaling in the IMQ group vs. the control group.

The IMQ model is a widely used murine model for the study of psoriasis like lesions in mice. IMQ is a ligand for Toll like receptors (TLR7 and TLR8) and when topically applied produces psoriasis-like skin lesions in mice which display many of the same characteristics as those observed in psoriasis in humans; elevations of IL-23/IL-17[15] and the dependency upon IL-22 to develop the lesions [20]. Thus, the IMQ model is an accepted model for psoriasis in mice. Recently it was shown that IMQ induced skin lesions in humans differs to some extent from native psoriasis plaques [21]. We compared the RNA microarray analysis from our study was to previously deposited array data by Swindell *et al*. [17]. This confirmed that the gene expression changes observed in our study were comparable to the previously deposited array data. Furthermore, genes that were differentially expressed between IMQ and IMQ-RSV mice were capable of perfectly separating samples from human psoriatic biopsies and normal healthy biopsies indicating a possible relevance in a human setting. Interestingly, using elements of the PASI we found that RSV could ameliorate the severity of erythema and scaling in the psoriasis-like condition. Using a calliper, these findings were confirmed by actual measurements of lesional skin showing significantly less thickening of the skin in the IMQ-RSV group compared to the IMQ group. Thus, a positive effect of RSV on the severity of the IMQ induced psoriasis-like skin condition was confirmed. In fact, RSV normalized the ear thickness in the IMQ-RSV group. Finally, the histological evaluation of lesional skin verified the finding that IMQ induces skin thickening and psoriasis-like infiltration of immune cells in the skin, and RSV primarily reduced thickness of the epidermal keratinocyte layer.

Ingenuity Analysis of the RNA microarray data revealed that several pathways were affected by RSV (Table 1). Interestingly, RXR signalling pathway was pinpointed, which might be important as retinoic acid (RA), a ligand for RXR, is a compound used in the treatment of psoriasis.

The RSV regulated genes from the RNA microarray analysis also revealed that TRIM63 expression was increased by RSV. TRIM63 is a member of the RING zinc finger protein, which has previously been implicated in the regulation of atrophy and hypertrophy (especially in striated muscle), by regulation of proteasomal degradation of proteins. Previous studies have demonstrated that glucocorticoid can activate TRIM63 in myoblasts [22] and simulated sun radiation, and UVB lighting activates TRIM63 in *in situ* human skin [23]. Both glucocorticoids and UVB are treatment modalities in psoriasis, and expression of the TRIM63 gene in skin from RSV treated mice may indicate a step towards atrophy, which in the case of IMQ induced thickening of the skin, may be beneficial.

In addition, we found that RSV increased gene expression of PCK1. This is interesting since both RA and glucocorticoids also stimulate PCK1. RA and glucocorticoids stimulate PCK1 gene expression in the liver as well as in adipocytes [24–26]. PCK1 is known to be regulated by FOX01 and RA controls PCK1 levels through FOX01 dependent pathways [27]. As RSV mediates at least some of its effects via FOX01 dependent pathways [28], we hypothesize that both RA as well as RSV stimulate PCK1 via FOX01. Another possible mechanism, by which RSV could regulate PCK1, is by affecting its acetylation status, as the degradation of PCK1 is controlled by the acetylation status of PCK1 [29]. RSV is a potent activator of SIRT1, which de-acetylates downstream targets like FOX01 [30] and other transcription factors that regulate PCK1 [31]. Therefore, it is possible that RSV regulates PCK1 through SIRT1 dependent regulation of the acetylation status of either PCK1 or important transcription factors.

The PPP1R3C, which is thought to control glycogen breakdown, was down regulated by RSV, lending support to our hypothesis that RSV might activate the RXR-pathway, since RA has been shown to down regulate PPP1R3C, albeit in another cell type [32].

The RNA microarray also identified IL-17A, IL-17F, IL-19 and IL-23p19 (a distinct subunit of IL-23) gene expression as being decreased by RSV. The ensuing Ingenuity Pathway analysis of the RSV effects among others highlighted several IL-17 dependent pathways as being changed significantly. This is interesting as IL-17A, IL-17F and IL-23 are major contributing factors in the formation of the psoriatic plaque both in humans and the model described by van der Fits *et al*. [15, 33] and IL-19 is known to potentiate effects of IL-17A [34]. IL19 which belongs to the IL-10 cytokine family has been shown to be upregulated in psoriatic lesions and IL19 induces keratinocyte growth factor production (KGF) [35]. As KGF signalling probably accounts for the epidermal hyperplasia associated with psoriasis [36] IL-19 activation might play an essential role in maintaining psoriasis plaques. Furthermore, IL-19 promoter is KGF responsive and therefore a positive feed-back loop may exist between IL-19 and KGF as reviewed by Gallagher [37]. Effective treatment of psoriasis is associated with a decrease in IL-19 level and already within one week of treatment with Etanercept (targeting TNFα signalling) IL-19 expression was rapidly decreased [38].

We performed qPCR to assess the IL-17A, IL-23p19 and IL-19 mRNA expression in the three groups of mice and found a significant increase in IL-17A, IL-23p19 and IL-19 expression in the IMQ treated group, compared with the control group. This is in accordance with the findings by van der Fits *et al*. [15] that the IMQ-induced psoriasis-like skin inflammation is critically dependent on signalling through the IL-23/IL-17 axis. RSV significantly decreased the IL-17A and IL-19 mRNA level in the lesional skin whereas IL-23p19 expression was not affected by RSV. The reason for this difference in responsiveness to RSV between the cytokines TNFα/IL-23 compared to IL-17/IL-19 is not completely understood. However as a previous study found that IL-19 mRNA level was more rapidly decreased after treatment with anti-TNFα than was IL-23 [38] our finding might be dependent upon the time after treatment. In addition our data could indicate that RSV may target IL-17 production which is a strong inducer of IL-19 [34].

Interestingly, IL-12/23 antibody targeting the p40 subunit, that is part of both interleukins 12 and 23, is already on the market for use against psoriasis. Recently, a new psoriasis treatment consisting of an anti-IL-17/anti-IL-17-receptor antibody has been developed with promising effects [39].However, there are some concerns about the side effects of the anti-IL17-receptor antibody, which approximately 70–80% of the test subjects experienced during the course of a 12 week clinical trial. Among side effects was neutropenia, which if severe, is a medical emergency because of increased risk of infection [40]. In light of that, a natural compound like RSV, which might increase the RXR gene expression and decrease IL-17A, IL-17F and IL-19 gene expression in psoriasis-affected skin, could be an interesting new treatment modality in human clinical trials. Particularly since RSV has already been given in high doses in clinical trials without any serious side effects; the most common side effect was abdominal discomfort occurring especially with doses above 2 grams per day [13, 41, 42].

In conclusion, RSV ameliorates the severity of IMQ induced psoriasis-like skin inflammation in mice. The skinfold thickening was reduced; the erythema and scaling scores improved and the histological analysis supported the calliper measurements and clinical findings of improvement. Using RNA microarray and Ingenuity Pathway analysis, we found that several interesting pathways were affected by RSV, like increased RXR expression and decreased expression of IL-17 dependent pathways as well as decreased expression of IL-17A, IL-17F and IL-19, and propose that these changes might be mediators of the positive effects observed on the psoriasis-like skin inflammation model used in our study. Comparison of the changes in gene expression in the mouse model to a human array dataset indicates that the observed RSV induced changes in the mice might play a role in the human setting. As RSV treatment in humans is well tolerated with no major side effects, RSV treatment of psoriasis patients might offer an interesting new treatment option. Based on our study, we encourage that pilot studies be performed to test if our results translate to the human setting and describe the efficacy and safety of RSV treatment in patients with psoriasis.

## Supporting Information

S1 Fig Cluster analysis. Example of cluster analysis.
(PDF)

S1 Table Primer sequences for PCR. Primers used for qPCR results and validation of microarray.
(PDF)

S2 Table RNA microarray and qPCR correlation analysis. Table is showing genes chosen for correlation analysis between the array ratio and the qPCR ratio. Raw data and ratios are provided. Ratios were used for Pearson Correlation calculation.
(PDF)
