# Supplementary figures and images for: Resveratrol Ameliorates Imiquimod-Induced Psoriasis-Like Skin Inflammation in Mice
Source: PLoS One. 2015 May 12;10(5):e0126599. doi: 10.1371/journal.pone.0126599 (PMC4428792; doi:10.1371/journal.pone.0126599)

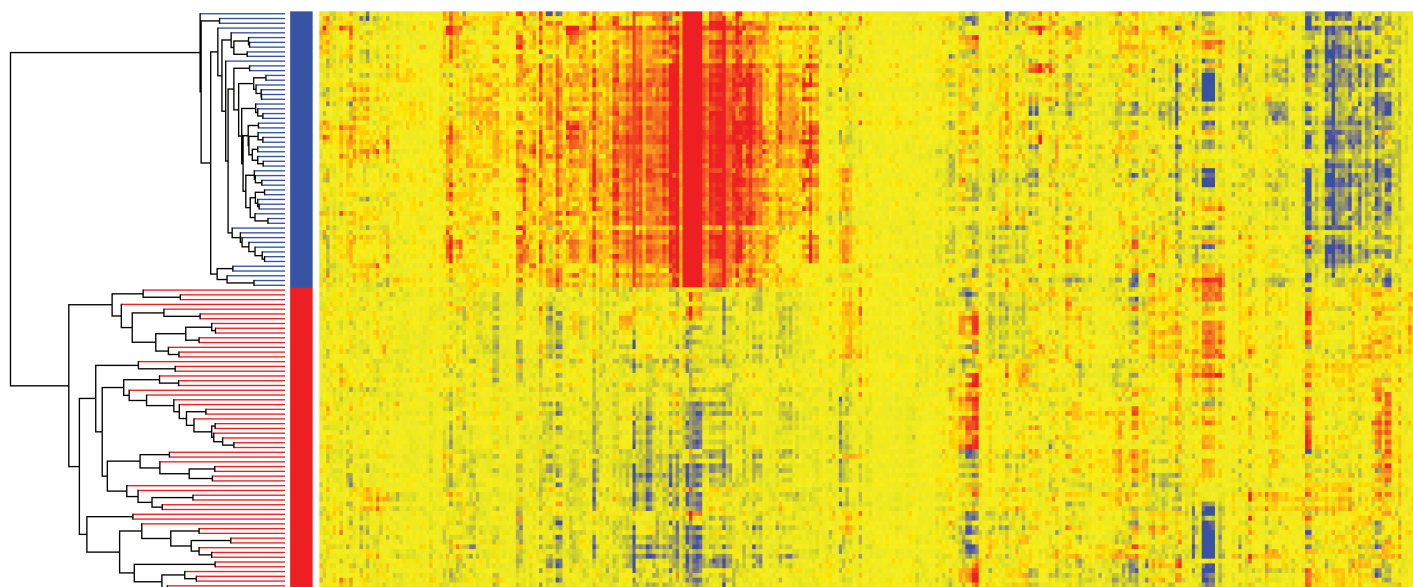

- Normal healthy controls
- Psoriatic patients

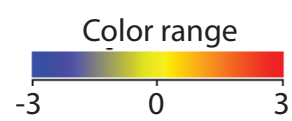

Supplement: S1 Fig — Example of cluster analysis. (PDF) [file pone.0126599.s001.pdf]
