# Supplementary material for: Resveratrol Ameliorates Imiquimod-Induced Psoriasis-Like Skin Inflammation in Mice
Source: PLoS One. 2015 May 12;10(5):e0126599. doi: 10.1371/journal.pone.0126599 (PMC4428792; doi:10.1371/journal.pone.0126599)
Supplement: S1 Table — Primers used for qPCR results and validation of microarray. (PDF) [file pone.0126599.s002.pdf]

S1 Table: Primer sequences for PCR

**Gene (M. musculus)**	**Forward primer**	**Reverse primer**
M. Alox8	AGGTGTCACCCTCTCTTCAAGC	GCAAGCGTGTTGATGTGCAGTG
M. Chi3l1	ATTACCAGGCCGGTTCACCAAG	CCTTTGAGGAAGTCGCATATCTCG
M.CXCL3	TGGTCAAGAAGTTTGCCTCAACCC	TGGACTTGCCGCTCTTCAGTATC
M. Defb14	CAGCTTGTTGGGAAGCAGTCAAC	AGCAGGCACCAAGAACAAGATGAG
M. Ear5	TGCCAGCACTTTGTACCTTCCC	AAGCCATATGCAGCGCAGACAG
M. Epgn	GCAGTCTGCCTCTTGTTCAAAGC	AGGGATCACCTCTGCTTCTTCG
M. Fam125a	AGAAAGCCGGCTACTTCCTGTG	ACCACATTGTCCTGAGGGTTCTC
M. Hbegf	TGGTGGCTGTAGTACTGTCGTC	CCTCCTCTCCTGTGGTACCTAAAC
M.Il-17a	TGAGTCCAGGGAGAGCTTCA	TTGGACACGCTGAGCTTTGAGG
M.Il-19	GGAGAACCTCAGGAGCATTAAGCC	AGAATGTCAGCAGGTTGTTGGTC
M.Il-33	CTGCCTCCCTGAGTACATACAATG	AGCGTAGTAGCACCTGGTCTTG
M.Lce1l	GTCCTCAGCGTTACCAGTTTCAGG	TGGCAGGACATTCTTGGGAGGAAG
M. Lce3e	GCCCTGCTGACTTCTTCTATCCAG	AGCTACCAGGGAATGAGGACTGTG
M. Lcn2	TTCACTCTGGGAAATATGCACAGG	GGCCACTTGCACATTGTAGCTC
M. Pck1	GCTGGATGTCGGAAGAGGACTTTG	GACATACATGGTGCGGCCTTTC
M. Ppp1r3c	ATGAGCTGCACCAGAATGATCC	GGCATGACGGAACTTGTCAAAGG
M. Psors1c2	AGCCATGATGCTCACCTGGAAG	TTGCCTGAAATGCCTCCTGCAC
M.PTGS2=COX2	TGGTGAAACTCTGGACAGACAAC	ATACACCTCTCCACCAATGACCTG
M. Serpinb3a	AGTATGATTGTCCTGTTGCCAGTG	AGCAGTGAGTTGTTCTTCAAGCTG
M. Sprr2b	GCAATTTCCACCATGCCAGCAG	GGATGGAAGTGTTGCTATGGAGTC
M. St6galnac5	CACAGGATGCTGCAGTTCGATG	CCAAGTGTTGGAGATCTTCCTGTC
M. Tmprss11g	GCGGGTACTTTGAAACGGCAAG	TGCTTGTGCTGCGTTCATTTCTC
M. Trim63	GGCCATTGACTTTGGGACAGATG	AGAGCGTGTCTCACTCATCTCC
M.Il-23	ACTCAAGGACAACAGCCAGTTC	GAAGATGTCAGAGTCAAGCAGGTG
M.TNFa	TTCCCAAATGGCCTCCCTCTCATC	TCCTCCACTTGGTGGTTTGCTAC
M. Myo18b[a]	GCACAAAGACCTCATTGCACAGTC	TGCAGCTGTAGCTCCTGAATCTG

Primers used for qPCR results and validation of microarray.

[a]Reference Gene
