# Supplementary material for: Resveratrol Ameliorates Imiquimod-Induced Psoriasis-Like Skin Inflammation in Mice
Source: PLoS One. 2015 May 12;10(5):e0126599. doi: 10.1371/journal.pone.0126599 (PMC4428792; doi:10.1371/journal.pone.0126599)
Supplement: S2 Table — Table is showing genes chosen for correlation analysis between the array ratio and the qPCR ratio. Raw data and ratios are provided. Ratios were used for Pearson Correlation calculation. (PDF) [file pone.0126599.s003.pdf]

S2 Table: RNA microarray and qPCR correlation analysis.

Gene	Control	IMQ Group	qPCR ratio	Control (unlogged)	IMQ Group (unlogged)	Array ratio
Alox8	0.03108	2.26111	72.751287	197.0999	2074.933666	10.52732
Chi3l1	0.0352	13.9946	397.5738636	382.4842	8264.131526	21.60646
CXCL3	0.016551	0.155788889	9.412413449	113.8574	119.1907527	1.046843
Defb14	0.21045	4.7067	22.36493229	229.5944	1759.570983	7.663825
ear5	0.2465	17.2321	69.90709939	236.9351	6450.787154	27.22596
Epgn	0.203063	2.1441	10.5588181	644.5633	1981.286777	3.073843
Fam125a	0.166125	1.21167	7.293724605	392.0928	360.7648573	0.920101
Hbegf	0.140525	3.6926	26.27717488	409.8124	1889.088401	4.609641
IL17	0.011501	0.11368	9.884143028	137.5364	161.2878777	1.172693
IL19	0.001685	0.65869	390.9139466	111.7653	539.7587816	4.829395
IL33	0.220488	2.3375	10.60150802	1026.205	2246.766528	2.189394
Lce1l	14.64963	38.645	2.637951483	1344.242	757.1922056	0.563286
Lce3e	0.0291	10.2981	353.8865979	74.15264	3165.620286	42.69059
Lcn2	0.114625	7.699	67.16684842	389.9534	3403.311524	8.727482
Pck1	0.138825	0.40761	2.936142626	420.6309	483.5043302	1.149474
Ppr1r3c	3.72175	4.1807	1.123315645	3440.863	1252.677216	0.364059
Psors1c2	1.15E-02	0.07735	6.72535594	142.4503	743.5232264	5.219528

PTGS2	0.079513	0.39032	4.908913693	636.7291	947.7419584	1.488454
Serpinb3a	0.104325	14.0302	134.485502	25.65687	1583.074297	61.70176
Sprr2b	0.445425	304.83	684.357636	119.408	7122.899897	59.65178
st6galnac5	0.041113	0.05758	1.400547279	287.3808	214.670509	0.74699
Tmorss11g	0.099988	3.4568	34.57232154	251.7448	1554.617019	6.175368
Trim63	0.540088	0.62616	1.159367695	1061.829	538.5190237	0.507162
Pearson Correlation						
R value	0.699679327					

Table is showing genes chosen for correlation analysis between the array ratio and the qPCR ratio. Raw data and ratios are provided. Ratios were used for Pearson Correlation calculation.
